# The Oral Cavity Potentially Serving as a Reservoir for SARS-CoV-2 but Not Necessarily Facilitating the Spread of COVID-19 in Dental Practice

**DOI:** 10.1055/s-0042-1757909

**Published:** 2022-12-20

**Authors:** Hironori Tsuchiya

**Affiliations:** 1 Department of Dental Basic Education, Asahi University School of Dentistry, Mizuho, Gifu, Japan

**Keywords:** oral cavity, SARS-CoV-2 reservoir, COVID-19 spread, dental practice, aerosol transmission

## Abstract

Intraoral tissues, secretions, and microenvironments may provide severe acute respiratory syndrome coronavirus 2 (SARS-CoV-2) with the conditions necessary for viral cellular entry and inhabitation. The aim of the present study is to overview the oral cavity that potentially serves as a reservoir for SARS-CoV-2, and then discuss the possibility that such oral cavity facilitates the spread of coronavirus disease 2019 (COVID-19) in dental practice. Articles were retrieved from PubMed/Medline, LitCovid, ProQuest, Google Scholar, and preprint medRxiv databases. Results of the literature search indicated that SARS-CoV-2 host cell entry-relevant receptor and virus/cell membrane fusion mediators are expressed in major and minor salivary glands, tongue, taste bud, periodontal tissue, and dental pulp, which would be a target and reservoir for SARS-CoV-2. SARS-CoV-2 is present in saliva and gingival crevicular fluid of COVID-19 patients. These secretions would contaminate dental aerosol and droplet with SARS-CoV-2. SARS-CoV-2 inhabits periodontal pocket, gingival sulcus, and dental caries lesion, which could provide SARS-CoV-2 with a habitat. SARS-CoV-2 ribonucleic acid is preserved in dental calculus, which may inform of the previous infection with SARS-CoV-2. Despite involvement of the oral cavity in SARS-CoV-2 transmission and infection, to date, there have been no clusters of COVID-19 in dental practice. Dental settings are much less likely to facilitate the spread of COVID-19 compared with general medical settings, which may be explained by the situation of dentistry that the number of patients to visit dental offices/clinics was decreased during the COVID-19 pandemic, the characteristics of dentistry that dental professionals have maintained high awareness of viral infection prevention, adhered to a strict protocol for infection control, and been using personal protective equipment for a long time, the experimental results that dental devices generate only small amounts of aerosol responsible for the airborne viral transmission, irrigant from the dental unit contributes to the aerosol microbiota much rather than saliva, and the commonly used evacuation or suction system effectively reduces aerosol and droplet generation, and the possibility that human saliva exhibits the antiviral activity and the property to inhibit SARS-CoV-2 infection. It is considered that dental treatment and oral health care can be delivered safely in the COVID-19 era.

## Introduction

Since the identification in Wuhan, China in late 2019, coronavirus disease 2019 (COVID-19) has spread worldwide with the continuous emergence of highly transmissible variants of its pathogenic agent, severe acute respiratory syndrome coronavirus 2 (SARS-CoV-2), reaching 590,420,628 cases with over 6.4 million deaths as of August 15, 2022 according to the Johns Hopkins University and Medicine Coronavirus Resource Center. 1 SARS-CoV-2 is commonly transmitted through close contact with COVID-19 patients coughing and talking without a mask, which results in inhalation of cough, sneeze, and respiratory droplet contaminated with the virus. Dental settings are presumed to possess additional risks of SARS-CoV-2 contagion, that is, working in proximity to patients, using tools and instruments contaminated with saliva and even blood that may contain the virus, and performing procedures to generate aerosol, droplet, and splatter that may be responsible for airborne virus transmission. 2 Nosocomial infection with SARS-CoV-2 and taking precautions against COVID-19 have been of great concern to dentistry. 3

The oral cavity is referred to not only as an entrance for exogenous pathogens to the human body but as a reservoir, source, or supplier for pathogenic agents of infectious diseases. Intraoral tissues, secretions, and microenvironments are expected to complete the requirements for SARS-CoV-2 entry into host cells, inhabitation, replication, and shedding. 4 There are increasing evidence to suggest that the oral cavity is associated with SARS-CoV-2 transmission and infection, and if so, dental professionals and patients are at a high risk of being exposed to SARS-CoV-2 during treatments.

Determining whether intraoral tissues, secretions, and microenvironments are suitable for SARS-CoV-2 cellular entry and inhabitation is important for recognizing that the oral cavity contributes to the efficient transmission of SARS-CoV-2 through dental procedures and for reducing the possibility that dental treatments would facilitate the spread of COVID-19. The aim of the present study is to overview the oral cavity that potentially serves as a reservoir for SARS-CoV-2, and then discuss the possibility that such oral cavity is closely associated with SARS-CoV-2 transmission and COVID-19 occurrence in dental practice or not.

## Methods

A literature search was performed in PubMed/Medline, LitCovid, ProQuest, and Google Scholar from April 1, 2021 with a cutoff date of June 30, 2022 by using the following terms or combinations thereof: “SARS-CoV-2 reservoir,” “oral cavity,” “oral tissue,” “oral secretion,” “COVID-19,” “dental practice,” and “aerosol transmission.” Given the rapid worldwide spread of SARS-CoV-2 infection and the ever-progressing studies on COVID-19, the preprint database medRxiv was also used to retrieve the most up-to-date information. The inclusion criteria were papers that were published in English more recently. Papers cited in the retrieved articles were further searched for additional references. Preprint articles, which had not been peer reviewed as of June 30, 2022, were excluded.

## Results and Discussion

The initial search resulted in 306 hits. After title and abstract screening, duplicate removal, and exclusion of articles not meeting the inclusion criteria, the remaining 82 studies underwent full-text review for overviewing the oral cavity to serve as a reservoir for SARS-CoV-2 and speculating its implications into the spread of COVID-19 in dental practice. Results of the literature search indicated that the oral cavity potentially serve as a reservoir, target, and/or source for SARS-CoV-2 as summarized in Table 1 .

**Table 1:** The oral cavity potentially serving as a reservoir for SARS-CoV-2

Intraoral tissue, secretion, or microenvironment	Possible association with SARS-CoV-2 transmission and infection	References
Salivary gland	ACE2, Furin, and TMPRSS2 are expressed in major and minor salivary glands. SARS-CoV-2 is detected in the salivary glands of COVID-19 patients. Salivary glands are infected with SARS-CoV-2, so they would secrete saliva containing SARS-CoV-2	Sakaguchi et al 8 Yoshimura et al 9 Zhu et al 10 Matuck et al 11 Huang et al 12
Saliva	SARS-CoV-2 is present in the saliva of symptomatic and asymptomatic COVID-19 patients, so saliva would contaminate dental aerosol and droplet with SARS-CoV-2	To et al 14 Tutuncu et al 15 Beyene et al 16 King et al 17 Chaudhary et al 18 Xu et al 19 Yang et al 20
Tongue	ACE2, Furin, and TMPRSS2 are expressed on the tongue, which would be infected with SARS-CoV-2	Sakaguchi et al 8 Xu et al 21
Taste bud	ACE2, Furin, and TMPRSS2 are expressed in taste receptor cell-containing taste buds, which would be infected with SARS-CoV-2	Sakaguchi et al 8 Park et al 22 Doyle et al 23
Oral mucosa (gingiva, palate, lip, and cheek)	ACE2, Furin, and TMPRSS2 are expressed in various oral mucosae, which could offer an infection route of SARS-CoV-2	Sakaguchi et al 8 Huang et al 12 Xu et al 21 Zhong et al 24 Almeida-da-Silva et al 25 Okui et al 26 Ohnishi et al 27
Periodontal tissue	ACE2, Furin, and TMPRSS are expressed in periodontal tissues. SARS-CoV-2 is detected in the periodontal tissues of COVID-19 patients. Periodontal tissues are infected with SARS-CoV-2, so they would supply SARS-CoV-2 to gingival crevicular fluid	Fernandes Matuck et al 28 Adam 29 Basso et al 31
Gingival crevicular fluid	SARS-CoV-2 is detected in the gingival crevicular fluid of COVID-19 patients. Gingival crevicular fluid would supply SARS-CoV-2 to gingival sulcus and pocket	Gupta et al 33
Gingival sulcus and periodontal pocket	Gingival crevicular fluid containing SARS-CoV-2 flows into gingival sulcus and periodontal pocket, which could provide SARS-CoV-2 with a habitat	Kheur et al 35 Elisetti 36 Natto et al 37 Badran et al 38
Dental caries lesion	SARS-CoV-2 is present in the cavitated caries lesions of COVID-19 patients	Natto et al 37
Dental pulp	ACE2 and TMPRSS2 are coexpressed in dental pulp, which would be infected with SARS-CoV-2	Galicia et al 39
Dental biofilm	SARS-CoV-2 RNA is detected in dental biofilms on the tooth surface of COVID-19 patients	Gomes et al 40
Dental calculus	SARS-CoV-2 RNA is preserved in dental calculus, which may serve as a reservoir of information on the previous viral infection	Berton et al 41

Abbreviations: ACE2, angiotensin-converting enzyme 2; COVID-19, coronavirus disease 2019; RNA, ribonucleic acid; SARS-CoV-2, severe acute respiratory syndrome coronavirus 2; TMPRSS2, transmembrane serine protease 2.

The mechanism underlying SARS-CoV-2 entry into host cells includes binding of the viral spike protein to a cellular receptor, angiotensin-converting enzyme 2 (ACE2), followed by viral and cellular membrane fusion that is mediated by cellular protein convertase Furin and transmembrane serine protease 2 (TMPRSS2). 5 The spike protein, one of four major structural proteins encoded by the SARS-CoV-2 genome, consists of two subunits: S1 responsible for binding to the host cell ACE2 and S2 for fusion of virus and host cell membranes. 6 The S1 subunit binds to the ACE2 receptor on host cells, and subsequently the spike protein is cleaved by Furin at the S1/S2 site to dissociate the S1 subunit. After the S1 dissociation, TMPRSS2 cleavage at the S2' site of the S2 subunit causes the fusion between the viral envelope and the host cell membrane, allowing SARS-CoV-2 to enter host cells. 7 Therefore, the expression of ACE2, Furin, and TMPRSS2 in intraoral tissues is an essential requisite for the oral cavity to be infected with SARS-CoV-2.

## The Oral Cavity as a Potential Reservoir for SARS-CoV-2

### Salivary Gland and Secretion

An immunohistochemical study of Sakaguchi et al indicated that ACE2, Furin, and TMPRSS2 are expressed in salivary glands of pathological samples collected from non-COVID-19 patients. 8 ACE2-positive cells are abundantly distributed in major (parotid and sublingual) and minor salivary glands as confirmed in biopsies from the patients with oral diseases morphologically and immunohistochemically. 9 ACE2 and TMPRSS2 are localized in the acinus and duct cells of human parotid, submandibular, and sublingual glands resected from patients who had been afflicted with benign disorders. 10 With respect to COVID-19 patients, Matuck et al conducted ultrasound-guided postmortem biopsies in COVID-19 fatal cases for immunohistochemical analysis. 11 They demonstrated that ACE2 and TMPRSS are intensively expressed in ductal epithelium and serous acinar cells of parotid and submandibular glands and minor salivary glands. In addition, their ultrastructural investigations showed that spherical viral particles consistent in size and shape with *Coronaviridae* family exist in the ductal lining cell cytoplasm, acinar cells, and ductal lumen of submandibular and parotid glands. SARS-CoV-2 infection of major (parotid and submandibular) and minor salivary glands was also elucidated by using autopsy tissues from COVID-19 patients. 12 SARS-CoV-2 infection with the subsequent exhibition of cytopathic effects on salivary glands causes oral symptoms associated with COVID-19 such as saliva secretory dysfunction 13 and sialadenitis/parotitis. 7 Given the expression of viral cellular entry-relevant biofactors and the detection of SARS-CoV-2 in COVID-19 patients, major and minor salivary glands are a target and reservoir for SARS-CoV-2. Such salivary glands can secrete saliva containing SARS-CoV-2 or contaminate saliva with SARS-CoV-2.

Since the detection of SARS-CoV-2 in saliva of COVID-19 patients, 14 the virus-contaminated saliva has been referred to as one of the causes of the nosocomial infection in dental offices/clinics. Tutuncu et al conducted reverse transcription-polymerase chain reaction (RT-PCR) analyses of saliva samples from COVID-19 patients as the cycle threshold (Ct) value from RT-PCR analysis is usable as a semiquantitative indicator of viral load. 15 SARS-CoV-2 positivity was observed in 90.6% of saliva obtained from mildly symptomatic and asymptomatic COVID-19 patients, who showed the mean Ct values of 28.36 ± 3.31 and 30.98 ± 2.39, respectively, being not different from those of nasopharyngeal specimens. When comparing different samples from symptomatic COVID-19 patients, 86% of the patients had higher viral loads in saliva than in nasopharyngeal swabs. 16 While low Ct values are associated with high viral load, the lowest Ct values were observed in saliva of patients infected with the Delta SARS-CoV-2 variant, followed by Alpha and Gamma, suggesting a relation between salivary viral load and transmissibility. 17 Chaudhary et al collected saliva from adult subjects with confirmed COVID-19 and without any COVID-19 symptoms. 18 In their RT-PCR experiments, salivary SARS-CoV-2 was detected in 23% of asymptomatic, 60% of postsymptomatic, and 28% of presymptomatic subjects. Even saliva of presymptomatic and paucisymptomatic patients potentially contributes to SARS-CoV-2 transmission because the viral ribonucleic acid (RNA) is detected in saliva before the appearance of lung lesions. 19 In a case of SARS-CoV-2 infection with the clinical course for more than 2 months, a high viral load was detected in saliva during the recovery period. 20 Saliva is responsible for SARS-CoV-2 transmission from symptomatic COVID-19 patients, asymptomatic patients in the incubation period of COVID-19, and COVID-19 survivors.

### Tongue and Taste Bud

Xu et al analyzed bulk RNA-seq profiles from public databases and suggested that ACE2 is expressed and enriched in human tongue epithelial cells. 21 Sakaguchi et al immunohistochemically confirmed that ACE2 and TMPRSS2 are coexpressed in the tongue epithelia of non-COVID-19 patients. 8 The tongue would serve as a target for SARS-CoV-2 and allow it to enter the human body.

Sakaguchi et al investigated the distribution of SARS-CoV-2 cellular entry-relevant biofactors in pathological tongue samples. 8 Their results indicated that ACE2, TMPRSS2, and Furin are localized in human taste buds containing taste receptor cells and taste bud-locating fungiform papillae. They also observed the expression of ACE2, TMPRSS2, and Furin in taste bud-derived culture cells. Park et al reported that ACE2 and TMPRSS2 are abundantly expressed in the squamous epithelia of tongue papillae, and ACE2- and TMPRSS2-positive cells are distributed in taste buds of the tongue. 22 ACE2 is specifically expressed in a subpopulation of taste receptor cells: the type II cells within taste buds located in human taste papillae that are infected with SARS-CoV-2. 23

Given the expression of SARS-CoV-2 cellular entry-relevant biofactors, the tongue and taste bud would be a target of SARS-CoV-2 and serve as a reservoir for SARS-CoV-2.

### Oral Mucosa (Gingiva, Palate, Lip, and Cheek)

The oral cavity mucosae are lined by stratified squamous epithelia as in gingiva and palate (keratinized mucosa) and in lip and cheek (non-keratinized). Xu et al analyzed public genomic databases and found that ACE2 is expressed in human gingival tissues. 21 Sakaguchi et al immunohistochemically revealed that ACE2, TMPRSS2, and Furin are expressed in gingivae of human pathological samples. 8 ACE2, TMPRSS2, and Furin expression are observed in the buccal surfaces of gingival epithelia. Huang et al performed the *in situ* hybridization mapping of oral tissue biopsies from healthy subjects. 12 They validated that ACE2 and TMPRSS2 are expressed in gingival, soft palatal, and buccal mucosa. Zhong et al collected normal mucosal tissues from human subjects, followed by the immunohistochemical staining to assess ACE2 and Furin expression. 24 Their results indicated that ACE2 and Furin are expressed in gingival, palatal, labial, and buccal mucosa. Western blots also showed the expression of ACE2 and TMPRSS2 in cultured gingival epithelial cells. 25 When comparing different sites of the oral cavity, the expression values of ACE2 were 14.6% for gingiva, 2.0% for palate, and 18.2% for tongue. 26 Ohnishi et al immunohistochemically stained human gingival tissues with or without periodontitis using an anti-TMPRSS2 antibody. 27 Gingiva with periodontitis was found to express TMPRSS2 more significantly than healthy gingiva. Periodontitis may make gingival tissues more susceptible to SARS-CoV-2 infection.

SARS-CoV-2 cellular entry-relevant biofactors are widely distributed in oral mucosae. Gingival, palatal, labial, and buccal mucosa could not only offer an infection route of SARS-CoV-2 but serve as a reservoir for SARS-CoV-2.

### Periodontal Tissue

SARS-CoV-2 was identified in periodontal tissues by postmortem biopsy and RT-PCR analysis in COVID-19 fatal cases. 28 SARS-CoV-2 is detectable in junctional epithelium, adjacent oral epithelium, and underlying connective tissue obtained from the mesial interproximal papillae of the maxillary first molar of COVID-19 patients. 29 While there is a correlation between COVID-19 and periodontitis, 30 ACE2, TMPRSS2, and Furin are expressed at a high level in periodontal tissues, especially in patients with periodontitis. 31 Proteases of periodontopathic bacteria have been speculated to cleave the spike protein of SARS-CoV-2 and enable the virus to enter target cells. 32

### Gingival Crevicular Fluid

Gingival crevicular fluid is a serum exudate from periodontal tissues, which contains components derived from the microvascular leakage. Gupta et al collected gingival crevicular fluid from the gingival sulci and periodontal pockets of mildly symptomatic and asymptomatic COVID-19 patients. 33 SARS-CoV-2 was detected in gingival crevicular fluid by RT-PCR analysis. Gingival crevicular fluid containing SARS-CoV-2 could contaminate saliva with the virus.

### Gingival Sulcus and Periodontal Pocket

Gingival crevicular fluid flows into gingival sulci and periodontal/gingival pockets. Therefore, SARS-CoV-2 contained in gingival crevicular fluid is able to migrate to gingival sulci and periodontal pockets as well as different viruses. 34 In addition, ACE2, TMPRSS2, and Furin are expressed in the gingival sulcular epithelium and periodontal pocket epithelium. 8

Gingival sulcus is a potential niche for SARS-CoV-2 and possesses the symbiotic relationship with local microbes. 35 There is a possible link between periodontal pocket and COVID-19. 36 Both periodontal pocket and gingival sulcus could provide SARS-CoV-2 with a habitat. When the swabs collected from the deepest pockets were subjected to RT-PCR analysis, SARS-CoV-2 was detected in the periodontal pockets of COVID-19 patients. 37 Periodontal pocket and gingival sulcus would serve as a reservoir for SARS-CoV-2. 38

### Dental Caries Lesion

Natto et al collected the swabs from cavitated caries lesions of COVID-19 patients to analyze SARS-CoV-2 E and S genes by RT-PCR. 37 They revealed that SARS-CoV-2 is present in the caries lesions, which could serve as a reservoir for SARS-CoV-2.

### Dental Pulp

In addition to pathogenic bacteria, certain viruses have been implicated in the pathogenesis of pulpitis. ACE2 and TMPRSS2 are coexpressed in biopsies of human normal and inflamed dental pulp tissues. 39 Dental pulp is considered to meet the requirement for SARS-CoV-2 infection and serve as a potential reservoir for SARS-CoV-2.

### Dental Biofilm

Various oral microorganisms form dental biofilm (immobile microbiome) that is composed of substances derived from saliva, gingival crevicular fluid, and microbial products. There are dental biofilms on the surfaces of tooth, hard and soft palate, and gingiva. Gomes et al collected dental biofilms from the buccal and lingual tooth surfaces of COVID-19 patients, followed by RT-PCR analysis. 40 The biofilms showed RT-PCR positivity for SARS-CoV-2.

### Dental Calculus

Berton et al conducted RT-PCR analyses of dental calculi collected from subjects who recovered from COVID-19 at the time of sample collection and from asymptomatic individuals in contact with mild COVID-19 patients. 41 SARS-CoV-2 RNA was detected in all samples, suggesting that dental calculus may serve as a reservoir of information on the previous infection with SARS-CoV-2.

### Intraoral Tissues Expressing Transient Receptor Potential Vanilloid Type 1

Since transient receptor potential (TRP) channels are expressed in various tissues infected with SARS-CoV-2, they may relate to SARS-CoV-2 infection. 42 Among the TRP superfamily, TRP vanilloid type 1 (TRPV-1) is known as a heat- and acid-activated ligand-gated nonselective cation channel that is specifically expressed in nociceptive sensory neurons. TRPV-1 contains ankyrin repeat domains as well as SARS-CoV-2 spike protein that contain two ankyrin-binding motifs. According to a study of Liviero et al, 43 TRPV-1 is responsible for the cellular binding of SARS-CoV-2.

There is increasing evidence that TRPV-1 is expressed in oral epithelia (tongue, gingiva, cheek, palate, and oral floor), 44 dental pulp, 45 periodontal tissue, 46 and salivary glands. 47 The oral cavity may serve as a target and reservoir for SARS-CoV-2 in association with intraoral TRPV-1 expression.

## SARS-CoV-2 Transmission and COVID-19 Spreading in Dental Practice

Dental practitioners must have the face-to-face communication with patients who may be in the incubation period of SARS-CoV-2 or have asymptomatic COVID-19. Dental treatments are performed in proximity to the oral cavity that potentially serves as a reservoir for SARS-CoV-2. Dentists, their team members, and patients are presumed to be at a high risk of SARS-CoV-2 transmission, especially when dental procedures generate a mixture of aerosol, droplet, and splatter contaminated with SARS-CoV-2. To date, however, there have been no confirmed cases of the spread of COVID-19 in dental offices/clinics or there have been very few SARS-CoV-2 transmission through dental procedures. 48 49 50 The clusters of COVID-19 were identified in hospital settings, whereas no clusters have yet been reported in dental settings of many countries. 51 52 SARS-CoV-2 transmission was observed neither from patients with COVID-19 to dental staffs nor from dental staffs with COVID-19 to patients in dental and oral/maxillofacial surgery departments of different university hospitals in Japan. 53 When a Web-based survey of dentists in the United States was conducted over a 6-month period, the cumulative prevalence rate of COVID-19 was 2.6% and the incidence rates ranged from 0.2% through 1.1% each month. 54 The occurrence rate of COVID-19 in dental practice is not different from that in the general population. 55 The question arises as to why the transmission of SARS-CoV-2 and the spread of COVID-19 are much less likely to occur in dental settings compared with general medical settings. Factors to affect the occurrence of COVID-19 associated with dental treatments are speculated as shown in Table 2 .

**Table 2:** Speculative factors to affect the occurrence of COVID-19 in dental practice

Factor	Contribution to reduction of SARS-CoV-2 transmission and COVID-19 spreading	References
Current situation of dentistry	The number of patients to visit dental offices/clinics was significantly decreased during the COVID-19 pandemic	Lee et al 56
Characteristic of dentistry	Dental professionals have maintained high awareness of viral infection prevention since the onset of HIV	Araujo et al 54 COVIDental Collaboration Group 55
Characteristic of dentistry	Dental professionals have adhered to a strict protocol for infection control since the raising awareness of hepatitis B and C infection	COVIDental Collaboration Group 55
Characteristic of dentistry	Dental professionals have been using personal protective equipment since long before the global COVID-19 outbreak	Barenghi et al 57
Efficacy of dental aerosol generation	The use of common dental devices generates only small amounts of aerosol responsible for the airborne viral transmission	Onoyama et al 64
Dental irrigant	Irrigant from the dental unit contributes to the aerosol microbiota much rather than saliva	Meethil et al 65
Commonly used evacuation or suction system	High-volume evacuator, extraoral vacuum aspirator, and extraoral suction device reduce the generation of dental aerosol, droplet, and splatter	Suwandi et al 66 Noordien et al 67
Potential ability of saliva	Human saliva exhibits the antiviral activity and the property to inhibit SARS-CoV-2 cellular entry and infection	Sakaguchi et al 8 Malamud et al 68 Drozdzik and Drozdzik 71 Tsukinoki et al 72

Abbreviations: COVID-19, coronavirus disease 2019; HIV, human immunodeficiency virus; SARS-CoV-2, severe acute respiratory syndrome coronavirus 2.

During the COVID-19 pandemic, dental treatment and oral health care were globally limited to urgent cases and nonemergency cases were postponed in accordance with international guidelines (Centers for Disease Control and Prevention [USA], European Centre for Disease Prevention and Control, etc.) and recommendations made by individual national dental associations. Consequently, the number of patients to visit dental offices/clinics has been significantly decreased. According to a report of Lee et al, 56 while the number of ambulatory patients was decreased in all hospitals, medical clinics, and dental clinics, the decrease of dental visits was much more drastic than that of medical visits. Such reduced utilization of dental services may have reduced the possibility of SARS-CoV-2 transmission and COVID-19 spreading in dental practice.

As suggested by the COVIDental Collaboration Group, 55 dental professionals have actively adopted preventive measures against viral infection since the onset of human immunodeficiency virus (HIV)/acquired immunodeficiency syndrome and the increased awareness of hepatitis B and C in the 1990s. Most of dental care providers have been continuing to abide by a strict protocol for infection control, which should help protect their patients and themselves from SARS-CoV-2 infection. 54 Dentists and their team members are in the habit of carrying out treatments based on the assumption that every patient visiting a dental office/clinic could be infected with HIV or hepatitis viruses. Since long before the global COVID-19 outbreak, they have been using personal protective equipment such as glove, face mask, face shield, filtering facepiece respirator, eye protector, protective gown, etc. in routine treatments. 57 These traditional characteristics of dental settings may explain the lower occurrence rate of SARS-CoV-2 transmission and the smaller number of COVID-19 cases compared with general hospital settings.

SARS-CoV-2 predominantly spreads through virus-contaminated aerosol (particles of ≤ 5 μm in diameter, the size enough to stay airborne), droplet (inspirable particles of > 5 μm in diameter), and splatter (particles of > 50 μm in diameter to behave in a ballistic manner) generated by dental treatment and oral surgery. 58 SARS-CoV-2 can be easily transmitted in enclosed environments because the aerosol and droplet contaminated with viruses travel up to 26 feet and keep the contagiousness up to 3 hours. 59 Nulty et al measured the amount of particulates (sized from ≤ 1 to 10 μm) generated during different restorative procedures and found that aerosol is significantly increased at the average working distance of dental clinicians. 60 Pierre-Bez et al assessed aerosol spread during ultrasonic scaling by determining droplet size and travel distance. 61 Consequently, observed particle sizes were consistent with those that can carry SARS-CoV-2, while aerosolized particles decreased as distance from the source increased. Any dental procedures to aerosolize the virus-containing intraoral secretions can cause airborne contamination with infectious pathogens. 62 The most common generation of aerosol, droplet, and splatter is attributed to the use of high-speed rotary handpiece, ultrasonic scaler, air abrasion device, and air-water syringe. 63 Dental aerosol and droplet would be contaminated with SARS-CoV-2 that is contained in intraoral secretions, especially saliva, and inhabits intraoral tissues and microenvironments.

Onoyama et al devised a through-cylinder system to mimic dental aerosol generation and assessed the particle size distribution of aerosol and droplet generated by using such devices as ultrasonic scaler, 3-way syringe, and dental engine. 64 Their laser diffraction analysis quantitatively demonstrated that all the tested devices generate only small amounts of aerosol smaller than 5 μm in diameter, and also suggested that the use of extraoral suction effectively prevents the spread of aerosol from high-speed dental engines. Meethil et al tracked the origins of microbiota in aerosol generated during ultrasonic scaling, implant osteotomy, and restorative procedures with high-speed handpieces by combining RT-PCR to quantify SARS-CoV-2 and 16S rRNA gene sequencing to characterize the entire microbiome. 65 They collected condensates from the face shields of operators and their assistants, the chests of patients, and the areas of 6-feet distance from the site of operation, unstimulated saliva from the subjects, and irrigant from the dental unit before the procedures, followed by identification of the source of microorganisms. It was proved that saliva does not necessarily contribute to the aerosol microbiota, whereas 78% of the condensate microbiota can be traced to the dental irrigant. SARS-CoV-2 was detected in the saliva of asymptomatic COVID-19 patients, but not in dental aerosol derived from them. The irrigant used in dental equipment, not the saliva from patients, is a major source of SARS-CoV-2. Aerosol-generating dental procedures are commonly accompanied by a dental evacuation, filtration, or suction system. Ultrasonic scaling simulation elucidated that a high-volume evacuator and an extraoral vacuum aspirator are so effective in reducing dental aerosol and droplet generation that they are useful for preventing SARS-CoV-2 transmission and infection. 66 When assessing the efficacy of different evacuation strategies in a clinical scenario, an extraoral aerosol suction device combined with a low-volume saliva ejector effectively decreased dental aerosol, droplet, and splatter. 67 It is considered that routine dental procedures may not necessarily be responsible for the airborne transmission of SARS-CoV-2.

Although saliva containing SARS-CoV-2 is referred to as a primary factor for the spread of COVID-19 in dental practice, saliva also contains bioactive components such as lactoferrin, agglutinin, cystatins, cathelcidin, histatins, and mucins, which potentially possess the antiviral activity. 68 They would affect the integrity and infectivity of SARS-CoV-2 in saliva as hyposalivation was suggested as a potential risk of SARS-CoV-2 infection. 69 Human saliva was reported to exert an inhibitory effect on Zika virus. 70 Instead of the direct viral inhibition, salivary immunity may be effective in inhibiting SARS-CoV-2 infection. Immunoglobulin A (IgA) could modulate SARS-CoV-2 infectivity because it is able to suppress the binding of the SARS-CoV-2 spike protein to the ACE2 receptor. 71 SARS-CoV-2 cross-reactive IgA is present in saliva of individuals who had never been infected with the virus, therefore salivary IgA would help prevent SARS-CoV-2 infection. 72 In addition, saliva contains protease inhibitors that may inhibit the activity of Furin and TMPRSS2, and RNase that may act as a resistance factor against RNA viruses. 8 These salivary components possibly inhibit SARS-CoV-2 cellular entry.

Although it is impossible to eliminate the risk of SARS-CoV-2 transmission through dental treatments completely, it is possible to minimize the risk of COVID-19 spreading in dental practice. A rubber dam is one of dental devices to reduce the viral load. The effective use of a rubber dam could eliminate virtually all microbial contaminations arising from saliva. 2 Although the scientific evidence remains to be elucidated, precautionary measures with mouth rinsing have been expected to prevent SARS-CoV-2 transmission. When COVID-19 patients rinsed their mouths with 1% hydrogen peroxide, 0.12% chlorhexidine, or 0.5% povidone-iodine for 60 seconds, the viral load in saliva was significantly decreased 15 and 45 minutes after rinsing. 18 The effects of 0.5% povidone-iodine and 0.075% cetylpyridinium chloride to decrease salivary viral load were reported to persist 6 hours after mouth rinsing. 73 Preprocedural mouth rinsing could reduce SARS-CoV-2 contamination of dental aerosol, minimizing the possibility of SARS-CoV-2 transmission from dental patients with asymptomatic and presymptomatic COVID-19.

If emergency dental care is needed for a patients suspected of SARS-CoV-2 infection, the treatment should probably be performed in an isolation room with negative pressure. Although negatively pressurized isolation rooms have been recognized to be effective for infection control and widely applied to COVID-19 patients, 74 the room incorporated with a high-efficiency ventilation and filtration system is not common in dental offices/clinics. As a low-cost complementary alternative, Teichert-Filho et al developed a device that consists of a rigid translucent acrylic structure designed to fit on the dental chair, covering the patient's head, neck, and chest regions, which is equipped with a piping system for the aspiration and filtration of air to provide a negative pressure inside the chamber. 75

## Conclusion

The oral cavity potentially serves as a reservoir for SARS-CoV-2. SARS-CoV-2 host cell entry-relevant biofactors, ACE2 receptor and viral cellular membrane fusion mediator Furin and TMPRSS2, are expressed and localized in major and minor salivary glands, tongue, taste bud, periodontal tissue, and dental pulp. Such intraoral tissues to be infected with SARS-CoV-2 would serve as a reservoir for SARS-CoV-2. SARS-CoV-2 is present in gingival sulcus, periodontal pocket, and cavitated caries lesion, which could meet the conditions for viral inhabitation. Saliva containing SARS-CoV-2 is presumed to play a critical role in viral transmission by contaminating dental aerosol, droplet, and splatter with the virus. However, routine dental procedures are much less likely to facilitate the nosocomial infection with SARS-CoV-2 and the spread of COVID-19 compared with general medical settings, which may be accounted for by the current situation of dentistry that the number of patients to visit dental offices/clinics was significantly decreased during the COVID-19 pandemic, the characteristics of dentistry that dental professionals have traditionally maintained high awareness of viral infection prevention, adhered to a strict protocol for infection control, and been using personal protective equipment since long before the COVID-19 outbreak, the experimental results that the use of dental devices generates only small amounts of aerosol responsible for the airborne viral transmission, irrigant from the dental unit significantly contributes to the aerosol microbiota much rather than saliva, and the commonly used dental evacuation or suction system effectively reduces aerosol, droplet, and splatter generation, and the possibility that human saliva exhibits the antiviral activity and the property to inhibit SARS-CoV-2 cellular entry and infection. Considering a high risk of SARS-CoV-2 transmission in dental offices/clinics, dental patients may refuse the necessary treatment and refrain from the regular checkup, which will adversely affect or give a negative impact on oral health. Fortunately, dental treatment and oral health care can be delivered safely even during the COVID-19 pandemic.
